# Clinical spectrum of congenital Zika virus infection in Brazil: Update and issues for research development

**DOI:** 10.1590/0037-8682-0153-2024

**Published:** 2024-07-29

**Authors:** Celina Maria Turchi Martelli, Fanny Cortes, Sinval Pinto Brandão-Filho, Marilia Dalva Turchi, Wayner Vieira de Souza, Thalia Velho Barreto de Araújo, Ricardo Arraes de Alencar Ximenes, Demócrito de Barros Miranda-Filho

**Affiliations:** 1 Instituto Aggeu Magalhães, Programa de Pós-Graduação em Saúde Pública, Recife, PE, Brasil.; 2 Universidade de Pernambuco, Pós-Graduação em Ciências da Saúde, Recife, PE, Brasil.; 3 Instituto Aggeu Magalhães, Departamento de Imunologia, Recife, PE, Brasil.; 4 Universidade Federal de Goiás, Programa de Pós-Graduação em Medicina Tropical e Saúde Pública, Goiânia, GO, Brasil.; 5 Universidade Federal de Pernambuco, Programa de Pós-Graduação em Saúde Coletiva, Recife, PE, Brasil.; 6 Universidade Federal de Pernambuco, Programa de Pós-Graduação em Medicina Tropical, Recife, PE, Brasil.

**Keywords:** Congenital Zika Syndrome, Congenital Zika Virus Infection, Review, Brazil

## Abstract

This review aimed to provide an update on the morphological and/or functional abnormalities related to congenital Zika virus (ZIKV) infection, based on primary data from studies conducted in Brazil since 2015. During the epidemic years (2015-2016), case series and pediatric cohort studies described several birth defects, including severe and/or disproportionate microcephaly, cranial bone overlap, skull collapse, congenital contractures (arthrogryposis and/or clubfoot), and visual and hearing abnormalities, as part of the spectrum of Congenital Zika Syndrome (CZS). Brain imaging abnormalities, mainly cortical atrophy, ventriculomegaly, and calcifications, serve as structural markers of CZS severity. Most case series and cohorts of microcephaly have reported the co-occurrence of epilepsy, dysphagia, orthopedic deformities, motor function impairment, cerebral palsy, and urological impairment. A previous large meta-analysis conducted in Brazil revealed that a confirmed ZIKV infection during pregnancy was associated with a 4% risk of microcephaly. Additionally, one-third of children showed at least one abnormality, predominantly identified in isolation. Studies examining antenatally ZIKV-exposed children without detectable abnormalities at birth reported conflicting neurodevelopmental results. Therefore, long-term follow-up studies involving pediatric cohorts with appropriate control groups are needed to address this knowledge gap. We recognize the crucial role of a national network of scientists collaborating with international research institutions in understanding the lifelong consequences of congenital ZIKV infection. Additionally, we highlight the need to provide sustainable resources for research and development to reduce the risk of future Zika outbreaks.

## INTRODUCTION

Approximately 10 years ago, an unprecedented outbreak of microcephaly occurred following the first Zika virus (ZIKV) epidemic wave in northeastern Brazil in 2015-2016. This outbreak has drawn global attention, highlighting the emerging threat posed by this arbovirus^1^ ^-^ ^3^. These clusters of severe microcephaly cases and other birth defects led to the discovery of the adverse effects of congenital Zika infection^4^ ^-^ ^6^. In early 2016, the World Health Organization declared a Public Health Emergency of International Concern (PHEIC) due to the increased frequency of neurological disorders and neonatal malformations^7^. ZIKV is a flavivirus primarily transmitted by *Aedes aegypti*, but can also be transmitted sexually and from mother to child^8^ ^,^ ^9^. ZIKV is an arbovirus that has recently been associated with teratogenicity^10^ ^,^ ^11^. Currently, Zika is classified as a priority disease with epidemic and PHEIC potential, along with Ebola, COVID-19 and others^12^.

In 2015, the Brazilian Ministry of Health implemented a new surveillance system to report cases of Congenital Zika Syndrome (CZS) and other infections among pregnant women and their offspring, in response to this public health emergency. This system is known as the Public Health Event Registry (RESP microcephaly)^13^ ^-^ ^15^. During the epidemic years (2015-2016), the peak prevalence of Zika-related microcephaly was 56.7 per 10,000 newborns in northeast Brazil. This marked a substantial increase compared with the pre-Zika era prevalence of 2.0 per 10,000 newborns^16^. A total of 1,858 cases were confirmed to have CZS between 2015 and epidemiological week 31 of 2023, with the majority identified during the epidemic years 2015-2016. After 2017, the number of CZS cases sharply declined, varying from 39 confirmed cases in 2018 to two in 2022 from various locations across the country. Despite this decline, a significant backlog of suspected CZS cases (n=2,960) remains under investigation, suggesting that the real burden may be underestimated^17^. Delays in diagnosing CZS are recognized as a surveillance pitfall^13^ ^,^ ^18^. Since 2019, CZS has been included in the priority surveillance of congenital anomalies at birth^18^. Brazil has documented dengue virus circulation in endemic and epidemic patterns since the 1980s^19^ ^,^ ^20^. The ongoing dengue epidemic in 2024 presents a current public health crisis^21^. Since 2015, the co-circulation of dengue, Zika, and Chikungunya, all transmitted by the same *Aedes aegypti* vector, suggests the persistent risk of these arboviruses within the country^21^ ^,^ ^22^. Therefore, Zika remains an epidemic threat, given its high vector density and large susceptible population nationwide.

CZS, now classified as a Congenital Zika virus infection (ICD11; KA62.0)^23^, has been associated with a wide range of fetal-neonatal adverse outcomes across various study settings^4^ ^,^ ^24^ ^-^ ^27^. These adverse outcomes have also been summarized in systematic reviews^28^ ^,^ ^29^. The mortality rates among cases with Zika-related microcephaly up to three years of life were significantly higher than those among non-microcephalic controls, according to official Brazilian datasets^30^. The long-term impacts of Zika infection in children and adolescents are still under investigation, underscoring the need for better understanding of the consequences of this infection throughout their lifespan.

This review provides an update of the spectrum of congenital Zika based on primary data from studies conducted in Brazil. We aimed to identify the morphological and/or functional abnormalities associated with congenital ZIKV infection throughout the life course and their impact on healthcare. Laboratory issues regarding Zika diagnosis and the pitfalls related to the co-circulation of other flaviviruses are beyond the scope of this update. Finally, we highlight the research priorities in this field. 

## SPECTRUM OF CONGENITAL ZIKA MANIFESTATIONS

We searched original articles describing the spectrum of congenital Zika manifestations using the PubMed database up to December 14, 2023. The following search terms were used: ((Zika OR ZIKV) AND (Congenital Abnormalities/classification OR Congenital Abnormalities/epidemiology OR Congenital Abnormalities/pathology) AND (Brazil)). We included primary data from studies specifying the morphological and/or functional abnormalities associated with the spectrum of congenital Zika, conducted in Brazil. Articles based exclusively on the results of surveillance-based analysis, laboratory findings, animal models, and modeling articles were excluded. We used the Rayyan application to screen titles and abstracts based on the eligibility criteria. A total of 296 publications were retrieved, of which 239 were excluded after abstract and title screening. Moreover, 57 articles were included in the review after full-text evaluation. We also screened the references of all included articles to identify additional studies.

During the epidemic years (2015-2016), the early descriptions of the clinical patterns of this newly recognized congenital infection included microcephaly, one of the most severe features of the spectrum. Other associated birth defects include disproportionate microcephaly, cranial bone overlap, skull collapse, congenital contractures (arthrogryposis and/or clubfoot), and visual and hearing abnormalities^24^ ^,^ ^31^ ^-^ ^37^. Although microcephaly is the predominant clinical feature of CZS, cases without microcephaly at birth have been also reported^38^. In addition, a previous study conducted in a pediatric cohort (n=23) followed from birth until 36 months reported the development of postnatal microcephaly, mainly in children with severe brain abnormalities at birth^39^. Brain imaging techniques such as computed tomography (CT) and magnetic resonance imaging (MRI) showed calcifications, ventriculomegaly, and diffuse cortical atrophy, which are now part of the diagnostic criteria^31^ ^,^ ^35^ ^,^ ^40^ ^-^ ^42^. A multicenter study of 83 children born with microcephaly at the peak of the epidemic, recruited from 10 Brazilian states, highlighted a phenotype known as the fetal brain disruption sequence, which is rarely observed in other congenital infections^43^.

A systematic review of systematic reviews (2015-2019) summarized the findings of 21 publications on health outcomes associated with ZIKV infection, mainly from Brazil, Colombia, and the United States. Microcephaly emerged as the most frequently reported feature of congenital ZIKV infection. The other reported sequelae during this period include fetal or neonatal death, intrauterine growth restriction, brain and eye abnormalities, and epilepsy^29^. Another systematic review summarized the profile of cases with CZS up to six months of life (2015-2019), focusing on anatomical and functional impairments^28^. The microcephaly and anatomical and functional alterations of the eye are the most common findings reported in the literature. Most of these microcephaly cases were born during the peak of the outbreak or shortly thereafter, with approximately half of the studies (27 of 46) originating from various regions of Brazil^28^.

Abnormal ophthalmological findings have been detected in children exposed to ZIKV with or without brain abnormalities^44^. The most common findings were optic nerve and retinal abnormalities, affecting 21.4% to 70% of the cases^45^ ^-^ ^52^. A multisite study (Pernambuco, Bahia, and Rio de Janeiro) analyzed the medical records of 468 infants with CZS with and without microcephaly. This study found that one-third of the cases developed ocular abnormalities, of whom 4% had no microcephaly^53^. The frequency of reported eye abnormalities may vary depending on the severity of the cases, age at the time of eye assessment, sensitivity and specificity of the ophthalmologic diagnosis (such as fundoscopy and retinography), and the lack of a standard protocol. To date, no study has compared the frequency of eye abnormalities between children born to mothers exposed and not exposed to ZIKV infection during pregnancy. Therefore, it remains unclear whether these figures are higher than those in unexposed children, which may be estimated based on relative risk. In addition, the attributable risk, which is the proportion of abnormalities among exposed children that may be attributed to exposure, remains unknown. Ophthalmological abnormalities have also been reported in other types of congenital infections^54^ ^,^ ^55^.

In a review of epilepsy and electroencephalography (EEG) patterns in CZS, the prevalence of epilepsy ranged from 37.7% to 71.4%^56^, depending on the severity of cases and time of follow-up. Epilepsy is considered a major neurological outcome of CZS in early infancy and has been observed in patients from different regions of Brazil^34^ ^,^ ^57^ ^-^ ^60^. In Pernambuco, a cumulative epilepsy incidence of 71.4% was observed in 91 cases with Zika-related microcephaly (MERG-PC) within the first two years of life. In addition, clinical data showed poor response to epilepsy treatment^61^. Consistent with the findings of previous studies, epileptic encephalopathy with spasm began after the third month of life^61^. In a 5-year follow-up study, Carvalho et al. (2023) highlighted changes in the longitudinal evolution of EEG and its relationship with brain imaging findings using CT or MRI^62^.

A systematic review of CZS and feeding disabilities in early childhood summarized 11 publications from different Brazilian settings. It highlighted that the frequency of dysphagia symptoms ranged from 17.9% to 70%^63^. Preliminary findings showed that nine infants with microcephaly over 3 months of age exhibited a significant loss of voluntary activity during the oral phase of swallowing^64^. A comprehensive evaluation of 116 children (MERG-PC), 50% of whom had microcephaly, found that oropharyngeal dysphagia was nine times more common among children with Zika-related microcephaly (79.3%) than in the normocephalic group (8.5%). The primary symptoms include coughing and choking during feeding, with approximately 20% of children with microcephaly requiring an alternative feeding route (such as a nasogastric tube or gastrostomy) at the age of 2 years. The microcephalic group also exhibited a higher frequency of hospitalization (41.4%) during the previous 6 months compared with the normocephalic group (16.2%)^65^. Dysphagia is currently recognized as a part of CZS and is linked to the severity of the spectrum.

Severe motor impairment is frequently observed in children with severe CZS. A systematic review conducted until March 2020 found that children with CZS present with severe motor impairment and a high frequency of spastic cerebral palsy within the first 2 years of life^66^. One study that included children with Zika-related microcephaly (n=59) showed that 81% (5-29 months) developed severe motor function impairment assessed based on the Gross Motor Function Classification System^67^. Another comprehensive study that included 75 preschool children with microcephaly found that distinct neurological profiles were associated with poor functional outcomes^58^. Moreover, several comorbidities, such as pneumonia and urinary tract infections, were highly prevalent among those with severe cases. The early onset of epilepsy, persistence of primitive reflexes, and dystonia are indicative of cortical hyperexcitability^58^. Neurological bladder conditions were also noted during follow-up^68^ ^-^ ^70^. Additionally, cases with a mean age of 40 months who developed Zika-related microcephaly (MERG-PC cohort) had endocrine dysfunctions such as early puberty, pubarche, adrenarche, hypothyroidism, short stature growth, and obesity^71^. Cryptorchidism has been reported in Zika-related severe microcephaly, warranting routine urogenital assessment and surgical intervention^72^ ^,^ ^73^.

Currently, substantial evidence indicates severe neurodevelopmental impairment in case series and cohort studies on CZS using different instruments. Eighty-nine children with CZS and cerebral palsy undergoing rehabilitation showed evidence of severe developmental delays at the age of 1 year, according to the Bayley Scale of Infant and Toddler Development III (BSID-III)^74^. A retrospective cohort study of 219 Zika exposed infants (53 microcephalic), aged 6-48 months, reported a high frequency of abnormal neurological outcomes assessed using BSID-III^75^. A large cross-sectional study of 274 children found a gradient of risk for neurodevelopmental delays, which was higher among children with severe or moderate microcephaly than among those without microcephaly who were born to ZIKV-infected mothers or neurotypical controls, using Survey of Wellbeing Young Children at two-years of age. Most children with severe microcephaly were classified “at risk of developmental delay”, compared with 65% of those with moderate microcephaly. Notably, similar and lower frequencies of alterations were found in prenatally ZIKV-infected cases and controls. The risk differences in these studies may be attributed to the degree of cerebral damage, indicating a poor prognosis in children with severe microcephaly^76^. Another study showed evidence of complete disability among children with Zika-related microcephaly assessed using the International Classification of Disability and Health^77^. During a 2-year follow-up study evaluating 42 microcephaly cases, major developmental delays were detected using HINE and BSID-III. However, this study highlighted heterogeneous development findings^78^. 

**FIGURE 1:**
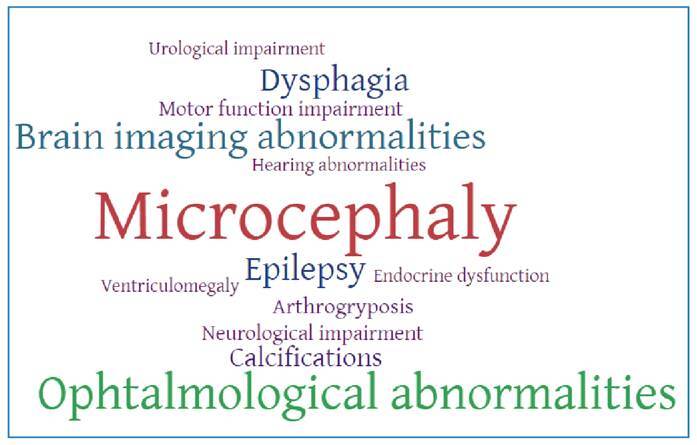
Word cloud generated from the frequency of adverse outcomes in the congenital Zika infection articles in Brazil.

In summary, Zika-related microcephaly, structural brain damage, such as cortical atrophy, ventriculomegaly, and calcifications, are part of the severe spectrum of CZS. Case series and pediatric cohort studies have described the co-occurrence of major adverse outcomes such as epilepsy, dysphagia, orthopedic deformities, motor function impairment, arthrogryposis, cerebral palsy, ophthalmological and hearing abnormalities, and urological impairment. Figure 1 shows a word cloud generated using the frequency of 141 cited words related to adverse outcomes from 44 abstracts of CZS-related publications. The size of the words in the cloud represents the frequency of the outcome in the abstracts. The most common adverse outcomes were microcephaly, ophthalmological abnormalities, brain imaging abnormalities, epilepsy, and dysphagia (Figure 1). This word cloud provides a helpful visual representation of the research findings in CZS based on the frequency of adverse outcomes cited in the published literature.

**Footnote:** The word size is proportional to the frequency of words related to adverse outcomes in abstracts.

## ANTENATAL ZIKV-EXPOSED CHILDREN WITHOUT DETECTABLE ABNORMALITIES AT BIRTH

A systematic review and meta-analysis included studies on children exposed in utero to ZIKV who did not show any abnormalities at birth. The pooled prevalence of neurodevelopmental delays were 6.5% for the non-language cognitive domain, 29.7% for the language domain, and 11.5% for the motor domain using the BSID-III^79^. Children without microcephaly who were born to women infected with ZIKV during pregnancy did not exhibit a significant increase in the risk of neurodevelopmental impairment in the first 42 months of life compared with their unexposed peers. However, a small group of children demonstrated a higher frequency of cognitive delay^80^. In a cross-sectional study conducted in Mato Grosso, no differences were found in cognitive, language, or motor development between non-microcephalic infants exposed to ZIKV and controls^81^. A large study conducted at Ribeirão Preto-São Paulo and São Luis-Maranhão showed that children who were exposed to ZIKV in utero did not show a higher risk of neurodevelopmental abnormalities compared with unexposed controls at 2 years of age, assessed using the BSID-III^82^. However, the results of different studies varied, indicating the need for standardized protocols and extended follow-up periods. 

## ESTIMATING THE ABSOLUTE RISK OF ADVERSE OUTCOMES

The Zika Brazilian Cohorts Consortium (ZBC-Consortium) is a data-sharing initiative that evaluated the absolute risk of adverse outcomes of congenital Zika. This project evaluated 13 pregnancy cohort studies in the Northeast, North, Southeast, and Central-West Brazilian regions, supported by the Brazilian Ministry of Health^27^. This individual participant data (IPD) meta-analysis included 1,548 ZIKV RT-PCR-positive pregnant women and their offspring. The overall absolute risk of microcephaly at 1-year follow-up was 4%. Additionally, 7.9% of the cases had central nervous system imaging alterations, 18.7% had neurological alterations, and 24.7% had at least one alteration. Isolated findings were more frequent than combined ones^27^. This risk gradient highlights the high frequency of isolated findings, which could eventually lead to later clinical manifestations that remain to be explored. One epidemiological question is whether the risk of Zika-related microcephaly is stable or variable in different settings^83^. The differences between countries and regions might be related to the stage of the epidemic, speed of the epidemic (variation in mosquito density, human population structure, density, and immunity), and the proportion of pregnant women infected during the first trimester of pregnancy^83^. The ZBC-Consortium data showed a similar risk of microcephaly across the four Brazilian regions^27^. Therefore, the results of this study suggest that the risk of Zika-related microcephaly is consistent across various settings. Furthermore, the similar frequency of adverse outcomes at multiple sites did not support the hypothesis of environmental and/or immune risk factors restricted to the northeast region.

Two other multi-country studies are ongoing: a cohort study of Zika in infants and pregnant women (ZIP study)^84^ and the World Health Organization IPD-meta-analysis^85^. A comprehensive metadata survey of 54 cohorts of Zika infected pregnant women supported by the WHO initiative showed heterogeneity in exposure and outcome ascertainment across the studies. This variability may be due to the lack of standard protocols and evolving knowledge in this field, considering the novelty of congenital disease^86^. Therefore, establishing a consensus on case and exposure definitions and harmonizing the variables across studies was the initial step of a time-consuming process that the researchers still faced to allow IPD-meta-analysis.

## FINAL COMMENTS

In general, microcephaly and abnormal brain imaging are consistently associated with the early prediction of poor neurological outcomes in different settings. These are the most severe manifestations, but are not the most frequent. The second group of children did not present with microcephaly but may have imaging, neurological, and/or other abnormalities, as evidenced by the ZBC-Consortium IPD meta-analysis. Finally, the third group of infants was exposed to ZIKV intra-utero without abnormal findings at birth; it remains under debate whether this group is more prone to neurodevelopmental delays. Studies with a proper control group, such as the meta-analyses currently being conducted by the ZBC and WHO consortia, will be essential for understanding the full spectrum of congenital Zika. This area requires further research in pediatric cohorts with prolonged follow-up.

As expected, most CZS clinical investigations were embedded in healthcare attendance at reference or specialized centers. In general, these diagnoses and follow-ups require multidisciplinary teams in pediatric subspecialties, such as infectious diseases, neurology, ophthalmology, endocrinology, audiology, pneumology, orthopedics, nutrition, urology, rehabilitative therapies, and family/caregiver support. These healthcare needs are complex and expensive, and are also encountered by patients with other congenital infections such as cytomegalovirus, toxoplasmosis, and rubella^25^. 

The WHO arbovirus initiative (WHO, 2020) aims to integrate mosquito-borne arbovirus strategies for Zika, dengue, and Chikungunya. The Zika virus poses a new challenge for arbovirus surveillance owing to its potential to cause adverse pregnancy outcomes. Currently, Zika prevention relies mainly on vector control and individual protection due to the lack of available vaccines and specific treatments. In this update, we acknowledge the outstanding role of the national network of scientists in collaboration with international research institutions in understanding the lifelong consequences of CZS. Therefore, sustainable resources for research and development must be provided to reduce the risk of future outbreaks.
